# Predictors of subjective memory functioning in young adults

**DOI:** 10.1186/s12888-025-06568-y

**Published:** 2025-02-19

**Authors:** Eszter Csábi, Fanni Mercédesz Kovács, Márta Volosin

**Affiliations:** 1 Department Of Cognitive and Neuropsychology, Institute of Psychology, University of Szeged, Egyetem utca 2, Szeged, H- 6722 Hungary; 2 Department of Psychiatry and Psychotherapy, Semmelweis University, Budapest, Hungary; 3 Institute of Cognitive Neuroscience and Psychology, Research Centre for Natural Sciences, Budapest, Hungary

**Keywords:** Subjective memory, Subjective memory complaints, Depression, Anxiety, Cognition, Sleep quality

## Abstract

**Background:**

The characteristics of subjective memory and the underlying cause of subjective memory complaints are well-established in the older population, but less is known about memory functioning and self-perceived memory deficiency in young adults. Therefore, we aimed to investigate the potential contributing factors of subjective memory complaints in young adults, such as objective cognitive performance, negative affective state, well-being and subjective sleep quality.

**Methods:**

125 participants over the age of 18 years were recruited in the study (average age was 21 years (SD = 2.29), 58 males and 67 females, average education of the participants was 14.1 years (SD = 1.94). We measured subjective memory with Multifactorial Memory Questionnaire and different cognitive domains (such as short- and long-term verbal and visual memory, working memory, and executive functions), negative affective states (depression and anxiety), well-being, and subjective sleep quality.

**Results:**

Our results showed that depression had a significant predictive value on satisfaction with memory and subjective memory complaints. We failed to find a relationship between perceived subjective memory functioning and objective cognitive performance. Besides anxiety, only the executive functions predicted significantly the frequency of internal strategy use.

**Conclusion:**

In conclusion, we revealed that negative affective states are more pronounced in the satisfaction with memory and subjective memory complaints in young adults than the objective cognitive ability.

## Introduction

Subjective memory is defined as how someone feels, thinks, or interprets their memory [1, 2]. This knowledge encompasses several aspects such as factual knowledge about tasks, memory strategies, and someone’s beliefs about their memory abilities; the perceived strengths and weaknesses in their everyday memory [3, 4]. These ingredients are essential in directing memory processes in overall decision-making [3, 5]. Subjective memory complaints are primarily relevant in cognitively normal older adults or patients with dementia or other neurological disorders but subjective memory complaints are also increasingly common among young adults. Examining self-perceived memory functioning, memory deficiency or strategy use in young adults may provide deeper insight into their learning strategies, problem-solving or performance at work or school, and overall self-efficacy [6]. Therefore, the goal of the current study is to investigate the contributing factors of subjective memory functioning such as objective cognitive performance, mood, well-being, and sleep quality in young adults.

A few studies focused on subjective memory functioning in the young population and these studies showed varying results. Some of them failed to find associations between subjective memory complaints and objective performance about episodic memory [6], and verbal, visual, and spatial components of working memory [7]. In contrast with these findings, Unworth and colleagues revealed that cognitive ability tasks, particularly attention control predicted subjective memory complaints and cognitive failures that can also predict scholastic abilities and general intelligence [8]. This result confirms those previous findings that individuals with high cognitive control experienced less memory failures [9, 10]. In line with these results, Crespo-Sanmiguel and colleagues found that subjective memory complaints related to episodic memory performance however, this negative relationship only appeared in participants with higher neuroticism [11].

Neuroticism is associated with affective traits such as anxiety and depression [12]. Based on previous literature, these negative affective states strongly affect subjective memory functioning in all age intervals [13]. Studies with young adults [4, 14, 15] demonstrated an association between subjective memory functioning and negative affect (depression and anxiety) underpinning Eysenck’s attention control theory that higher level of negative emotions reduces attention focus and results more subjective memory complains [16, 17]. Utilizing Multifactorial Memory Questionnaire (MMQ) [18], depression and anxiety were differently associated with subjective memory functioning depending on age. While depression predicted satisfaction with memory irrespectively of age, it was associated with self-rated ability in older adults only. On the other hand, anxiety predicted memory satisfaction and self-rated memory ability both in the younger and older adults [19]. Furthermore, the severity of subjective memory problems strongly correlated not only with affective status but also with well-being [6, 20, 21].

In addition to objective cognitive performance and mood, sleep quality is also an important factor contributing to subjective memory functioning [19]. Insufficient sleep is common in young adults due to biological changes (e.g. circadian rhythm), psychosocial challenges (e.g., academic requirements, social relations) or electric media use [22]. A recent study by de Zambotti and colleagues reported that the prevalence of inadequate sleep (e.g., short sleep duration, insomnia, difficulties in falling asleep, staying asleep) in adolescents and young adults is ranging between 4 and 39% [23]. Healthy sleep is essential for cognitive functions and emotion regulation, therefore poor sleep quality may contribute to altered cognitive functioning and emotional dysregulation [24, 25].

Previous studies on subjective sleep quality and cognitive performance have led to controversial findings. Some studies found a relationship between sleep quality and attention, executive function [24], or decision-making [26], while others suggested the lack of association between sleep quality and cognitive functions [27, 28]. It is important to note, that these studies examined objective cognitive performance, and not subjective memory. To the best of our knowledge, two studies so far investigated the effect of sleep quality and sleep duration on subjective memory performance by using MMQ in young adults. The study by Ling and colleagues found an association between insomnia symptoms but not objective sleep duration and self-perceived memory functioning. Participants with insomnia were less satisfied with their subjective memory functioning indicating that sleep restriction influences on perceived subjective memory [29]. Furthermore, Rekers and colleagues demonstrated that inadequate sleep predicted memory satisfaction in middle-aged adults, and self-rated memory ability in young and middle-aged adults [19].

Due to the small number of studies with controversial findings in young adults, the present study aimed to investigate the potential contributing factors of subjective memory complaints in young adults such as objective cognitive performance, negative affective state, well-being and subjective sleep quality. As the vast majority of the studies presented above examined the relationship between these constructs and subjective memory functions *separately* (but see [19]), less is known about the additive effect of these factors. In order to investigate this question, we applied hierarchical linear regression models allowing to add sets of predictors in a sequential manner [30]. The outcome variables were the MMQ subscales, and potential predictor variables were added in three levels. At the first level, objective performance was included because it is featured by manifold aspects and it can be considered as the most commonly studied factor in association with subjective memory. At the second level, we added affective variables which are also often investigated in the context of metamemory. Finally, sleep-related variables were added as only a few studies targeted the association between sleep and subjective memory functioning.

We hypothesised that subjective memory functioning is determined more by negative affective state [4, 14, 15, 19], well-being [20, 21] and subjective sleep quality [19, 29] than objective cognitive performance in general [7, 8]. Alternatively, we also expected that if there is an association with any of the objective tasks, it will be present in tasks measuring executive functioning [8–10].

## Methods

### Participants

139 participants over the age of 18 were recruited into the study via university courses, email and social media. The mean age of the participants was 21.78 years (SD = 4.50), range = 18–54 years, 63 males and 76 females. The average education of the participants was 14.24 years (SD = 2.09; range = 12–20 years). The inclusion criteria of the study were the followings: age between 18 and 28 years, free of any medication that can affect sleep or cognitive functioning and no cognitive impairments. After excluding persons not meeting the inclusion criteria, 125 participants remained. The mean age of the participants was 21 years (SD = 2.29), range = 18–28 years, 58 males and 67 females. The average education of the participants was 14.1 years (SD = 1.94), a range of 12–20 years. Before the experiment, all participants provided informed consent. The study was conducted in accordance with the Declaration of Helsinki and the protocol was approved by the United Ethical Review Committee for Research in Psychology, Hungary (EPKEB; Reference number: 2024-068).

## Tasks

### Subjective and objective memory tasks

#### Multifactorial Memory Questionnaire (MMQ)

MMQ is a self-report questionnaire designed to assess different aspects of self-perceived memory functioning [18]. The Hungarian version of the questionnaire consists of four subscales [21]. *Satisfaction* subscale contains 18 items that measure someone’s satisfaction with memory functioning. *Ability* subscale consists of 22 items that assess the most common memory problems over the previous two weeks. *External strategy* subscale contains 7 items that measure the frequency of external compensatory strategies in everyday life, such as “make a list”, or “use a timer or alarm”. The *Internal strategy* subscale composed of 10 items, indicates the frequency of internal strategies such as “create an image” or “create a rhyme”. These two subscales indicate only the frequency of use and not the reason for using the aids [18]. Note that although– similar to the original tool– the Hungarian version of MMQ contains 57 items, only 55 items are used for calculating scale scores [21]. Participants have to rate each item on a 5-point Likert scale (1 = never, 2 = rarely, 3 = sometimes, 4 = often, 5 = always). Individuals with higher scores on the subscales are more satisfied with their memory, have better subjective impressions about their memory capacities, and use more external and internal memory strategies [18, 21].

#### Rivermead Behavioral Memory Task (RBMT)

We used three subtests from RBMT to measure short- and long-term verbal and visual memory performance. The subtests were the following: (1) *Remembering a name*: a portrait was presented to the participants along with the first name and surname of the person on the picture. The subject has to repeat the names immediately (immediate recall) and 20 min later (delayed recall). Two points were given for the correct answer and one point if only one part of the name matched. (2) *Remembering a newspaper article*: a short prose passage was read to the participants and afterward they have to recall the story immediately (immediate recall) and 20 min later (delayed recall). One point was given for each correctly recalled information. (3) *Picture recognition*: 10 line-drawing pictures of common objects were shown to the subjects and asked to recognize them among a set of 20 pictures (original 10 and 10 distractors) (immediate recall). After a 20-minute delay, the participants had to recognize the original 10 pictures again out of 20 pictures (delayed recall). One point was given for each correctly identified picture [31, 32].

#### Word list

To measure short-term verbal memory, we used a word list. In this task, the participants were asked to listen and recall an increasingly longer list of words. It starts with four words; if they can recall correctly, we increase the number of words. The maximum length is 8 words. The participant’s verbal memory span is the longest list they can remember [33].

#### Listening Span

The working memory performance was measured by the Listening Span task. In this test, participants are instructed to listen and verify increasingly longer sequences of sentences and to recall the final word of all the sentences in each sequence in serial order. A participant’s working memory capacity is defined as the longest sequence length at which they are able to recall the final words [34, 35].

#### Digit Span

Digit Span task is designed to assess verbal short-term and working memory. In this task, participants are asked to repeat sequences of increasing number of digits exactly as in the order presented. The number of digits in each sequence increases from 3 to 9. The total score corresponds to the maximum number of digits the participant can repeat correctly [36, 37].

### Executive functions tasks

#### Wisconsin Card Sorting Task (WCST)

Wisconsin Card Sorting Task was used to measure executive functions. In this task, four stimulus cards are displayed on the computer screen. The participant receives two sets of response cards, and they have to categorize them according to color, shape, and number. The participant is instructed to match each of the response cards to one of the stimulus cards [38, 39]. After the participant’s code was registered to the custom-made program, the “The task is starting” text was displayed on the screen, then after 300 ms the first trial was presented. In each trial, one response card was presented at the bottom of the screen and five stimulus cards were presented at the top of the screen. Participant had to choose one of the stimulus cards matching a given dimension (color, shape or number) of the response card by clicking with mouse. Each trial remained on the screen until the participant responded, that is, no time pressure was introduced. After each trial, participant got a visual (“Right choice!” or “Oops! Wrong choice!”) feedback whether they were right or wrong. The feedback remained on the screen for 1000 ms, then after a 300 ms blank screen the next trial was presented. The task consisted of 120 trials. Responses faster than 100 ms and slower than 5000 ms were excluded from the further analysis. Participants were characterized with the percentage of correct responses, the number of perseveration errors, and the median of the reaction times separately for correct and incorrect responses.

#### Letter and Semantic Fluency Tasks

We used Letter and Semantic Fluency tasks to measure executive functions. During these tasks, participants have to generate as many words as they can within 60 s with restrictions that cannot be the listed word’s proper nouns, numbers, and multiple forms of the same root word. In the Letter Fluency task, the subjects have to list words beginning with a specified letter; in the Semantic fluency task people have to generate words from a specified category. In this study, we used letter “k” for letter fluency and the “animals” category for semantic fluency. We registered the number of correct words, the perseverations, and errors in both fluency tasks [40, 41].

### Depression, anxiety, and subjective well-being

#### Patient Health Questionnaire-9 (PHQ-9)

The PHQ-9 is a 9-item self-reported questionnaire designed to evaluate the presence of depressive symptoms during the prior 2 weeks. The participants have to answer on a 4-point Likert scale about the frequency of the occurrence of each symptom (0 = not at all to 3 = nearly every day) and have to rate the severity of the symptoms (caused difficulties in work, household, or social relations). Higher scores indicate a higher level of depressive symptoms [42].

#### Beck Anxiety Inventory (BAI)

BAI is a self-administered questionnaire of the most common symptoms of anxiety. The questionnaire consists of 21 items; each item is rated on a 4-point Likert scale. The participants have to indicate how much they have been bothered by the symptoms during the past month [43, 44].

#### World Health Organization Five Well-being Index (WHO-5)

WHO-5 is a 5-item rating scale used to assess current mental well-being. The participants have to rate the perceived frequency of each item in the past two weeks on a 4-point Likert scale (0 = at no time to 3 = all of the time). Higher scores represent higher well-being [45, 46].

### Subjective sleep quality, sleepiness, and insomnia

#### Pittsburgh Sleep Quality Index (PSQI)

PSQI is a self-administered questionnaire used to measure subjective sleep quality. It is composed of 19 items with the following subscales: perceived sleep quality, sleep latency, sleep duration, sleep efficiency, sleep disturbances, hypnotic medication use, and daytime dysfunctions. Each subscale received a score based on a Likert scale of 0 to 3, where 0 reflected “not during the past month” and 3 indicated “three or more times per week”. The global score ranges between 0 and 21, and higher scores indicate worse sleep quality [47, 48].

#### Van Dream Anxiety Scale (VDAS)

VDAS is designed to capture dream anxiety during the preceding month. The questionnaire contains 17 items about sleep duration, sleep quality, and the frequency and consequences of nightmares (e.g., fear of sleeping, difficulty falling asleep after a nightmare, morning anxiety, memory or concentration problems, sleepiness, occupational-, familial-, and social distress). The responders are required to answer each item on a 4-point Likert scale, where 0 reflects ‘never’ and 4 reflects ‘very often”. The total score ranges between 0 and 42, higher scores indicate a higher level of dream anxiety [49, 50].

#### Groningen Sleep Quality Scale (GSQS)

We used the Groningen Sleep Quality Scale to assess the sleep quality of the night prior testing. This self-reported questionnaire consists of 15 items and the responders have circled the true or false for each question. GSQS scores range from 0 to 14, with higher scores indicating lower subjective quality of sleep [51, 52].

#### Epworth Sleepiness Scale (ESS)

ESS is a self-administered questionnaire designed to measure excessive daytime sleepiness. It contains 8 situations and the responders have to rate on a 4-point Likert scale their tendency to become sleepy (0 = no chance of dozing to 3 = high chance of dozing). The total score is based on a scale of 0 to 24, higher scores indicate a higher level of daytime sleepiness [53].

#### Athen Insomnia Scale (AIS)

We used AIS to measure the symptoms of insomnia. This self-reported questionnaire contains 8 items that ask about the difficulties of falling asleep, night and morning awakenings, total sleep time, the quality of sleep, and the next-day consequences of insomnia (overall functioning and sleepiness during the day). The participants are requested to rate each item on a 3-point Likert scale (0 = no problem at all to 3 = very serious problem). The total score on the questionnaire is 24 points, higher scores indicate a higher level of insomnia [54].

### Procedure

Participants over the age of 18 years were recruited via university courses, email, and social media. If they met with the inclusion criteria, they were summoned to the Laboratory of the Institute of Psychology, University of Szeged to carry out the objective cognitive tasks. Before the objective testing, the participants received the informed consent and the questionnaires by email and were asked to complete them the day before the evaluation session. The questionnaires took about 20 min to fill out and the laboratory assessment took approximately an hour.

### Statistical analysis

In the first step of the statistical analysis, Spearman correlations were calculated between the four subscales of MMQ as well as the objective cognitive tests, affectivity scales and questionnaires on sleeping. Because the large number of correlations (*n* = 31) would lead to the enhanced probability of Type I errors, Bonferroni correction was applied and alpha level was corrected to 0.05/31 = 0.0016 [55]. Accordingly, only correlations were considered as statistically significant with *p* < 0.0016.

In the second step, hierarchical linear regression models were built with Enter method where the outcome variables were the subscales of MMQ, and the predictors were the significantly correlating variables with these subjective scales after Bonferroni correction. We applied hierarchical regression models because they allow to measure constructs not only by individual predictors but also by the combined contribution of sequentially added sets of predictors [30].

We decided to select the predictors based on their correlation with the outcome variables because of the following reasons. First, the present study uses relatively lots of potential predictors, and when the number of predictors is higher than necessary for the proper description of the data, it might lead to noise, therefore selecting a subset of variables is recommended [56]. Second, this two-step approach is also applied in other studies on metamemory (e.g., [57, 58]).

The hierarchical regression models were built as follows. At the first level, the scores of objective tests, at the second level the scores of affective variables and at the third level, sleep-related variables were submitted as predictors. When no significant correlations were present regarding a certain level, the hierarchical regression consisted of fewer than three levels. For each model, Durbin-Watson (DW) test was run to define the level of autocorrelation, and multicollinearity was assessed by variation inflation index (VIF) for the predictors. VIF was considered to be as satisfactory below 3 [59], and DW values were satisfactory between 1.5 and 2.5. Statistical analysis was conducted in jamovi (version 2.2.5) [60].

## Results

### MMQ satisfaction

Regarding objective cognitive tests, no significant p values were present after Bonferroni correction, but significant negative moderate correlations were found with PHQ-9 (*r*_*s*_(123) = -0.444, *p* < 0.001), BAI (*r*_*s*_ (123) = -0.458, *p* < 0.001), AIS (*r*_*s*_(123) = -0.355, *p* < 0.001) and with PSQI Subjective Sleep Quality (*r*_*s*_(123) = -0.326).

Because of the lack of significant correlations with the objective tests, the hierarchical linear regression model had only two levels: the first contained PHQ-9 and BAI scores and the second contained AIS and PSQI Subjective Sleep Quality scores as predictors. The Durbin-Watson test revealed the lack of autocorrelation (Model 1: 1.890 and Model 2: 1.923). In Model 1, predictors explained 14.9% of the variance (*R*^*2*^_*adj*_ = 13.5%; *F*(2, 120) = 10.519, *p* < 0.001) and Model 2 explained 17.7% of the variance (*R*^*2*^_*adj*_ = 14.9%; *F*(4, 118) = 6.333, *p* < 0.001). However, the difference between the two models was not significant (*ΔR*^*2*^ = 2.8%, *F*(2, 118) = 1.976, *p* = 0.143), and while in Model 1 PHQ-9 had significant predictive value (*β* = -0.257), it disappeared as sleep-related variables were entered to the model (*β* = -0.181). This suggests that entering sleep-related variables did not improve the model, moreover they even weakened the effect of depression. The results of the models are presented in Table 1.

**Table 1:** Results of the hierarchical linear regression model where the outcome variable is MMQ satisfaction score

		B	SE(B)	Β	t	*p*	VIF
Model 1	Constant	60.270	2.442		24.678	< 0.001	
	PHQ-9	-0.725	0.304	-0.257	-2.390	0.018	1.635
	BAI	-0.249	0.159	-0.169	-1.573	0.118	1.635
Model 2	Constant	61.113	2.529		24.164	< 0.001	
	PHQ-9	-0.511	0.361	-0.181	-1.416	0.159	2.350
	BAI	-0.269	0.158	-0.183	-1.697	0.092	1.659
	AIS	-0.078	0.549	0.019	0.142	0.887	2.474
	PSQI	-3.627	2.054	-0.191	0.080	0.080	1.673

Note: PHQ-9 = Patient Health Questionnaire-9; BAI = Beck Anxiety Index; AIS = Athens Insomnia Scale; PSQI = Pittsburgh Sleep Quality Index

### MMQ ability

No significant correlations were present after Bonferroni correction either with objective tests of sleep-related variables. Only one moderate negative correlation was found with PHQ-9 (*r*_*s*_(123) = -0.289, *p* = 0.001). Therefore, only a single level linear regression model was run where the only predictor was PHQ-9 value. The Durbin-Watson value was satisfactory (1.627). The model was significant, the PHQ-9 explained 7.1% of the variance (*R*^*2*^_*adj*_ = 6.3%; *F*(1, 122) = 9.313, *p* = 0.003). The results of the model are presented in Table 2.

**Table 2:** Results of the linear regression model where the outcome variable is MMQ ability score

		B	SE(B)	β	t	*p*	VIF
Model 1	Constant	60.570	2.320		26.106	< 0.001	
	PHQ-9	-0.692	0.227	-0.266	-3.052	0.003	1.000

Note: PHQ-9 = Patient Health Questionnaire-9

### MMQ external strategy

No objective, affective or sleep-related data showed significant correlation with MMQ External Strategies, indicating that the use of external strategy is not influenced by objective cognitive performance, affective state or sleep quality.

### MMQ internal strategy

After Bonferroni correction, significant positive moderate correlations were present between MMQ Internal Strategy subscale and the number of items produced on Letter fluency task *r*_*s*_(123) = 0.301, *p* < 0.001 and between BAI scores (*r*_*s*_(123) = 0.325, *p* < 0.001). No significant relationship was found with any of sleep-related variables.

Therefore, at the first level of the hierarchical linear regression, the result of Letter fluency task was submitted, and at the second level BAI was entered into the model. In Model 1, predictors explained 6.3% of the variance (*R*^*2*^_*adj*_ = 5.5%; *F*(1, 123) = 8.219, *p* = 0.005) and Model 2 explained 16.4% of the variance (*R*^*2*^_*adj*_ = 15%; *F*(2, 122) = 11.966, *p* < 0.001). The inclusion of the affective scales significantly improved the predictive value of Model 2 compared to Model 1: *ΔR*^*2*^ = 10.1%, *F*(1, 122) = 14.792, *p* < 0.001. The value of Durbin-Watson test was 2.039 for Model 1 and 2.058 for Model 2, suggesting the lack of autocorrelation. In the Model 1, Letter fluency performance significantly predicted the use of internal strategy (*β* = 0.250). In Model 2 when BAI score was entered, both Letter fluency (*β* = 0.228); and BAI (*β* = 0.319) revealed to be significant predictors. The results of the models are presented in Table 3.

**Table 3:** Results of the hierarchical linear regression model where the outcome variable is MMQ Internal Strategy score

		B	SE(B)	β	t	*P*	VIF
Model 1	Constant	3.557	2.010		1.670	0.097	
	Letter fluency	0.309	0.108	0.250	2.867	0.005	1.000
Model 2	Constant	1.743	1.951		0.894	0.373	
	Letter fluency	0.281	0.102	0.228	2.746	0.007	1.005
	BAI	0.216	0.056	0.319	3.846	< 0.001	1.005

Note: Letter fluency = the number of correct items produced on Letter fluency task; BAI = Beck Anxiety Index

## Discussion

The aim of the current study was to examine the contributing factors of subjective memory complaints in young adults. Our results showed that satisfaction with memory functioning (MMQ– Satisfaction subscale) exhibited a negative correlation with depression, insomnia, and subjective sleep quality. Depression had a significant predictive value on memory satisfaction. Still, it disappeared as sleep-related variables were entered into the model, indicating that sleep parameters such as insomnia and subjective sleep quality weakened the effect of depression. We failed to find a relationship between objective cognitive performance and memory satisfaction. MMQ– Ability subscale that assesses the frequency of most common memory problems also showed a lack of correlation with objective cognitive performance, but it was associated negatively with depression. The external strategy use (MMQ– External Strategy subscale) did not show any correlation with depression, anxiety, objective performance, or any of the sleep-related variables. In contrast, internal strategy use (MMQ– Internal Strategy subscale) demonstrated a significant relationship with Letter Fluency Task and anxiety, indicating that executive functions and the level of anxiety predicted the internal strategy use.

We revealed that depressive symptoms associated with memory satisfaction and the frequency of memory problems, indicating that individuals with higher level of depression were less satisfied with memory functioning and exhibited more memory complaints. These results corresponded to those previous studies that demonstrated a negative association between negative affect and subjective memory complaints in young adults [4, 14, 15, 19]. A study by Jensen and colleagues using MMQ found a negative correlation in young adults between depression, memory satisfaction, and ability, while external and internal strategies showed a positive association with depression scores [4]. Some studies supposed that the relationship between subjective memory concern and negative affective state exists irrespective of age [13, 19, 21], although others have found the opposite conclusion and assumed that subjective memory concerns are more frequent in older adults [4, 19, 61]. These studies focusing on older adults demonstrated that subjective memory evaluations relate to affective states rather than cognitive abilities where there is no cognitive decline [62, 63]. However, it is still unknown whether memory problems or concern about cognitive decline can cause anxiety and depression or whether the negative affective state has an influence on subjective memory functioning [13, 64–66]. A study by Jonker et al. [67] concluded that the lack of cognitive impairment in young adults suggests that the influence of negative affective states is greater than in older participants.

Our findings with young adults are in line with Eysenck and colleague’s attention control theory which states that anxiety disrupts the attention control system and can lead to more frequent memory mistakes [16]. There is also evidence that stress exposure that can be associated with anxiety has a negative impact on everyday memory [14]. Another explanation might be that negative affect is associated with enhanced self-monitoring and awareness of errors [68], and these traits might cause greater reported subjective memory complaints and frequent use of internal strategies [21]. Parallel with this observation, we also found in the current study that higher level of anxiety was positively correlated with the frequency of internal strategy use. Previous studies with older adults also emphasized that the perceived memory failures and negative affect would be more pronounced in subjects with high levels of anxiety [15, 69]. Tandem with these findings, it is also possible that individuals with a higher level of anxiety have an inaccurate perception of their memory abilities, along with higher vulnerability and worse coping strategies in stressful situations [70, 71].

The role of sleep in cognitive functioning has not been fully delineated. Some studies revealed that sleep restriction has a negative impact on working memory, episodic memory [72], or problem-solving [73]. In line with these results, our data showed that individuals with better subjective sleep quality and fewer insomnia symptoms are more satisfied with their memory. These results are on a pair with Rekers and colleagues [19] who found a link between sleep quality and self-rated memory ability in young and middle-aged adults. In addition, consistent with these findings, Ling and colleagues [29] also demonstrated the negative effects of insomnia symptoms but not the objectively measured sleep duration on subjective memory, proposing that sleep quality is related to perceived memory functioning instead of sleep quantity. In contrast with our results, other studies failed to find an association between sleep and cognitive ability [27, 28, 74], but it is important to highlight that these experiments used objective cognitive tasks only. To better understand the possible effect of sleep on cognition future studies should also examine objective and subjective memory and their relationship with objective measures of sleep, for example with polysomnography.

Similarly to the results with younger population, studies examining the relationship between subjective memory functioning and objective cognitive abilities in the older population showed varied results. Some of them revealed an association with cognitive performance [75–78], while others failed to find a correlation between objective and subjective measurement [62, 63]. The possible reasons for the inconsistent results may be due to the characteristics of the examined population (e.g. age, gender, education, clinical sample) [65, 79, 80] or methodological differences between the studies (see [81]). Our results are in tune with the previous literature showing that subjective memory concerns are not related to objective cognitive performance in young adults [1, 7]. These results might be explained by the fact that the participants did not exhibit enough memory difficulties to express high memory complaints [11]. Another possible explanation is that there are more distractions in everyday life, therefore individuals have to multitask and divide their attention between tasks compared to laboratory settings when they have to focus on one task at the same time. Consistent with this hypothesis, objective and subjective measurements may assess different types of memory [82]. While questionnaires evaluate past experiences, general ability in everyday life, and information about memory capability and stereotypes, objective tasks often have learning materials to be tested later, thus its asses learning ability in laboratory conditions that may not necessarily reflect real-life situations [80, 83–85]. Furthermore, the use of external memory aids is usually not allowed in laboratory settings, while it can be useful in daily life contexts [65]. Contrary to our findings, Crespo-Sanmiguel and colleagues found a relationship between objective and subjective memory performance but only in participants with higher levels of neuroticism [11]. Furthermore, Unworth and colleagues revealed that participants who demonstrated poor attention control in laboratory conditions experienced more everyday attention failures. The authors pointed out that the level of cognitive control, particularly attention control can predict objective and subjective memory performance [8]. Attention control skills are especially important in situations where there are numerous of external (e.g. noisy environment) and internal distractions (worrying about something) [8].

Previous studies demonstrated differences between age groups in subjective memory functioning, and the differences often might be explained with situational, habitual or environmental factors. For example, Mogle et al. [66] found that older adults reported more retrospective memory lapses than younger adults. Others showed that age correlated negatively with the satisfaction of memory and strategies indicating that young adults are more satisfied with their memory and utilize more strategies (particularly internal strategies), but they failed to find a relationship with memory abilities [4, 86]. The authors suggest that these results may in part be explained by the fact that all the young adults in their sample were university students at the time of testing and were probably more likely to use mnemonic strategies to help their academic performance compared to older adults and non-students [4]. Contrary to these results, Radnan et al. [87] findings indicated that external strategies were more prevalent than internal strategies for both age groups. Older adults reported more strategies overall and were more likely to report physical and environmental tools than digital tools which are more likely to be used by young adults [87]. There is also evidence that stereotypes can negatively affect the evaluation of memory even if objective memory performance is within a normal range [18, 65, 88]. Older adults expect to achieve lower results, leading them to be unmotivated and subsequently lower performance [89]. In addition, older adults may think that cognitive decline is unavoidable and attribute their daily memory failures to cognitive decline. Like those with cognitive difficulties who are unable to assess accurately their memory abilities and tend to underestimate or overestimate them [65, 82, 90]. Furthermore, confounding memory with other cognitive functions can lead in overreporting problems such as memory difficulties [79, 82]. In contrast, young adults may think that everyday memory mistakes are insignificant or more reversible with more control over their abilities [91]. In line with these findings, older adults may underestimate their memory abilities while younger adults overestimate their memory capability [65, 92]. In addition, young and middle-aged adults may assess their memory abilities more accurately than older adults because they can update their self-evaluation through feedback from work or education [93, 94]. From the other point of view, older adults may be more aware of everyday memory failures due to general expectation of memory decline, and therefore be more accurate in their memory evaluation [65, 80, 93, 94]. Based on these controversial findings, further investigation needs to clarify these discrepancies in different age groups and the underlying mechanism between objective cognitive ability and subjective memory performance.

Our results also showed that executive functions were associated with the frequency of internal strategy use representing that higher level of executive functioning is linked to more internal strategy use. Some previous studies also revealed a positive relationship between subjective memory complaints and prefrontal symptoms [95–97]. Molina-Rodrigez and colleagues reported that executive functions and perceived stress together explained 57% of the subjective memory complaints [96]. In their study, attention and executive control problems had the greatest weight in the model. Additionally, executive control and attentional problems showed a mediating effect between perceived stress and subjective memory complaints [96].

Executive functions are responsible for higher-order human functioning including switching and inhibitory control of attention [98, 99]. They “reflect an individual’s capacity to maintain information, including task goals, in a highly active state despite interference. It’s keeping relevant information highly active and easily accessible reflects an individual’s ability to control attention, because coherent and goal-oriented behaviour in interference-rich conditions requires both the active maintenance of relevant information and the blocking or inhibiting of irrelevant information” (pp. 170) [100, 101]. Based on this theoretical underpinning, our findings might be explained by individuals with higher levels of executive control using more internal strategies to avoid subjective memory failures. This hypothesis also highlights the critical role of attentional control in everyday memory function just as in Unsworth and colleagues’ experiment [8]. In the older population, some studies also found a negative relationship between executive functioning and subjective memory problems [75, 77]. Based on the compensatory theory, executive functioning and effective compensatory strategies mediate the association between subjective memory complaints and objective cognitive performance indicating that better executive abilities are related to less subjective memory failures and lower rates of objective performance [79].

This finding raises the question of why individuals do not use less capacity-intensive external strategies instead of internal strategies. We assumed that the possible reason for the lack of correlation between external strategy use and executive control is that MMQ was originally designed to measure the older population’s subjective memory complaints, therefore mainly asking about strategies such as “write things on a calendar”, and “use a timer or alarm” or “write down in a notebook”. In our study, we examined young adults who are more likely to use digital tools (e.g., smartphones) rather than physical external compensatory strategies [87]. However, we cannot rule out the possibility that we still get the same results even if the questionnaire contains questions about digital reminders. It is also possible that young adults with higher executive functions might feel more confident about their memory functioning and managing their daily tasks [102], therefore they might not feel the need to use additional external strategies besides internal strategy.

Our study has potential limitations that should be considered. Firstly, we used MMQ originally designed for measuring subjective memory in the older adults, therefore it might be not sensitive enough in the younger population [4], however, our results and some previous studies found that young adults have similar symptoms and predictors or subjective memory mistakes as individuals in older age [6]. Future studies might be worth adding further questions about the use of digital tools, not only in young population but also in older ages, due to the increasing use and positive attitude about digital technology in older adults [87]. Secondly, objective and subjective testing was conducted on two separate times two weeks apart, which may have caused the lack of correlations between subjective and objective measurement; however, two weeks may not be a long time for a significant change in participants. For future studies, it is worth testing participants in one session with pauses. Furthermore, previous studies suggest that neuroticism has been associated negatively with lower levels of cognitive functioning such as episodic and working memory [11, 103] or executive functions [104] that could lead to more memory mistakes. Moreover, mind-wandering is also related to neuroticism which can disrupt the concentration on the present moment resulting in more perceived memory failures [105, 106]. In our study we evaluated only the negative affective state, therefore future studies need to focus on these personality traits to fully address this question. Finally, it is also important to note the drawback of our statistical method, namely instead of including all potential predictors into the hierarchical regression models, we have selected only a subset of them. Although selecting predictors by different methods is a commonly used approach to reduce noise [56], keeping the excluded variables still might have contributing predictive value to the outcome variables which remained undetected this way. In addition, as hierarchical regression is suitable for detecting direct effect of predictors only [30], our results might serve as starting point for future studies to identify mediator or moderator variables related to subjective memory complaints, objective performance, affect and sleep.

## Conclusion

In conclusion, we found a similar pattern in young adults as in previous studies in older adults [62, 63]. Our findings suggest that negative affective states such as depression and anxiety are more pronounced in the satisfaction with memory and subjective memory functioning than cognitive ability. These results underline the importance of screening affective status when examining memory at all ages and might provide additional information about the characteristics of subjective memory and the possible causes of subjective memory concerns in young adults that might help to develop training programs for young adults to improve work, academic achievement or overall self-efficacy in everyday life. Furthermore, subjective memory is a multidimensional construct and self-report evaluation reveals significant individual differences and information about subjective memory concerns. Therefore, our findings confirm those previous studies [18, 107] that emphasize that the best option would be to provide the most accurate memory profile to combine objective and subjective measures in research and clinical practice.

## Data Availability

All data, analysis details, and research materials are available at https://osf.io/t7yjr/.

## Abbreviations

AIS: Athen Insomnia Scale
BAI: Beck Anxiety Inventory
DW: Durbin-Watson test
ESS: Epworth Sleepiness Scale
GSQS: Groningen Sleep Quality Scale
MMQ: Multifactorial Memory Questionnaire
PHQ-9: Patient Health Questionnaire-9
PSQI: Pittsburgh Sleep Quality Index
RBMT: Rivermead Behavioral Memory Task
SD: Standard Deviation
VDAS: Van Dream Anxiety Scale
VIF: Variation Inflation Index
WCST: Wisconsin Card Sorting Task
WHO-5: World Health Organization Five Well-being Index

## References

[1] Pearman A, Storandt M. Predictors of subjective memory in older adults. J Geront: Ser B. 2004;59(1):P4–6. 10.1093/geronb/59.1.p4 14722332

[2] Troyer AK, Leach L, Vandermorri S, Rich JB. The measurement of participant-reported memory across diverse populations and settings: a systematic review and meta-analysis of the multifactorial memory questionnaire. Memory. 2019;27(7):931–42. 31020904 10.1080/09658211.2019.1608255

[3] Pannu JK, Kazniak AW. Metamemory experiments in neurologic populations: a review. Neuropsychol Rev. 2005;15(3):105–30. 16328731 10.1007/s11065-005-7091-6

[4] Jensen A, Castro AW, Hu R, Drouin H, Rabipour S, Bégin-Galarneau MÉ, Stamenova V, Davidson PSR. Multifactorial memory questionnaire: a comparison of young and older adults. Memory. 2024;32(8):1043–56. 39018424 10.1080/09658211.2024.2378870

[5] Szajer J, Murphy C. Education level predicts retrospective metamemory accuracy in healthy aging and Alzheimer’s disease. J Clin Exp Psychol. 2013;35(9):971–82. 10.1080/13803395.2013.844771 PMC3909664 24131064

[6] Pearman A. Predictors of subjective memory in young adults. J Adult Dev. 2009;16:101–7.

[7] Wright DB, Osborne JE, Dissociation. Cognitive failures, and Working Memory. Am J Psychol. 2005;118(1):103–14. 15822612

[8] Unsworth N, Brewer GA, Spillers GJ. Variation in cognitive failures: an individual differences investigation of everyday attention and memory failures. J Mem Lang. 2012;67(1):1–16. 10.1037/a0028075 22468805

[9] Engle RW, Kane MJ. Executive attention, working memory capacity, and a two-factor theory of cognitive control. In: Ross B, editor. The psychology of learning and motivation. New York: Elsevier; 2004. pp. 145–99.

[10] Unsworth N, Spillers GJ. Working memory capacity: attention, memory, or both? A direct test of the dual-component model. J Mem Lang. 2010;62:392–406.

[11] Crespo-Sanmiguel I, Zapater-Fajarí M, Garrido-Chaves R, Hidalgo V, Salvador A. Subjective memory complaints in young people; their relationship with objective cognitive performance and the role of neuroticism. Annals Psychol/ Anales De Psicol. 2024;40(2):323–34.

[12] Barlow DH, Curreri AJ, Woodard LS. Neuroticism and disorders of emotion: a New Synthesis. Curr Direct Psychol Sci. 2021;30(5):410–7.

[13] Rowell SF, Green JS, Teachman BA, Salthouse TA. Age does not matter: memory complaints are related to negative affect throughout adulthood. Aging Ment Heal. 2016;20(12):1255–63. 10.1080/13607863.2015.1078284 PMC5135579 26305735

[14] Molina-Rodriguez S, Pellicer-Porcar O, Mirete-Fructuoso M, Martinez-Amoros E. Subjective memory complaints, perceived stress and coping strategies in young adults. Revista Neurol. 2016;62(8):344–50. 27064913

[15] Zapater-Fajarí M, Crespo-Sanmiguel I, Perez V, Hidalgo V, Salvador A. Subjective memory complaints in young people: the role of resilience. Psychol Health. 2022;39(9):1243–62. 36368933 10.1080/08870446.2022.2141240

[16] Eysenck MW, Derakshan MN, Santos R, Calvo MG. Anxiety and cognitive performance: Attentional Control Theory. Emotion. 2007;7(2):336–53. 17516812 10.1037/1528-3542.7.2.336

[17] Eysenck MW, Jason S, Derakshan MN, Hepsomali P, Allen P. A neurocognitive account of attentional control theory: how does trait anxiety affect the brain’s attentional networks? Cogn Emot. 2023;37(2):220–37. 36583855 10.1080/02699931.2022.2159936

[18] Troyer AK, Rich JB. Psychometric properties of a new metamemory questionnaire for older adults. J Geront: Psychol Sci. 2002;57(1):19–27. 10.1093/geronb/57.1.p19 11773220

[19] Rekers S, Heine J, Thöne-Otto AIT, Finke C. Neuropsychiatric symptoms and metamemory across the life span: psychometric properties of the German multifactorial memory questionnaire (MMQ). J Neurol. 2024;271:4551–65. 38717611 10.1007/s00415-024-12402-4 PMC11233313

[20] Derouesne´ C, Lacomblez L, Thibault S, LePoncin M. Memory complaints in young and elderly subjects. Int J Ger Psych. 1999;14:291–301. 10340191

[21] Csábi E, Hallgató E, Volosin M. The association between metamemory, subjective memory complaints, mood, and well-being: the Hungarian validation of multifactorial memory questionnaire. Cogn Res: Princip Impl. 2023;8:15. 10.1186/s41235-023-00469-y PMC9928992 36786909

[22] Owens J. Insufficient sleep in adolescents and young adults: an update on causes and consequences. Pediatrics. 2014;134(3):e921–32. 25157012 10.1542/peds.2014-1696 PMC8194472

[23] de Zambotti M, Goldstone A, Colrain IM, Baker FC. Insomnia disorder in adolescence: diagnosis, impact, and treatment. Sleep Med Rev. 2018;39:12–24. 28974427 10.1016/j.smrv.2017.06.009 PMC5931364

[24] Benitez A, Gunstad J. Poor Sleep Quality diminishes cognitive Functioning Independent of Depression and anxiety in healthy young adults. Clin Neuropsychologist. 2012;26(2):214–23. 10.1080/13854046.2012.658439 22335237

[25] O’Leary K, Bylsma LM, Rottenberg J. Why might poor sleep quality lead to depression? A role for emotion regulation. Cogn Emot. 2016;31(8):1698–706. 27807996 10.1080/02699931.2016.1247035 PMC6190702

[26] Telzer EH, Fuligni AJ, Lieberman MD, Galván A. The effects of poor quality of sleep on brain function and risk taking in adolescence. NeuroImage. 2013;71:275–83. 23376698 10.1016/j.neuroimage.2013.01.025 PMC3865864

[27] Németh D, Janacsek K, Londe Z, Ullman MT, Howard DV, Howard JH Jr. Sleep has no critical role in implicit motor sequence learning in young and old adults. Exp Brain Res. 2010;201:351–8. 19795111 10.1007/s00221-009-2024-x

[28] Zavecz Z, Nagy T, Galkó A, Németh D, Janacsek K. The relationship between subjective sleep quality and cognitive performance in healthy young adults: evidence from three empirical studies. Scient Rep. 2020;10:4855. 10.1038/s41598-020-61627-6 PMC7078271 32184462

[29] Ling J, Sun W, Chan NY, Zhang J, Lam SP, Li AM, Chan JWY, Kyle SD, Xin Li S. Effects of Insomnia symptoms and objective short sleep duration on memory performance in youths. J Sleep Res. 2020;29(4):e13049. 32394606 10.1111/jsr.13049

[30] Hoyt WT, Imel ZE, Chan F. Multiple regression and correlation techniques: recent controversies and best practices. Rehabil Psychol. 2008;53(3):321–39.

[31] Kónya A, Racsmány M, Czigler B, Takó E, Tariska P. Introduction of the rivermead behavioural memory test (RBMT). Hung J Psychol. 2001;55(4):435–49.

[32] Wilson BA, Cockburn J, Baddeley A. Rivermead behavioural memory test. Thames Valley Test Company; 1985.

[33] Németh D, Ivády RE, Miháltz M, Krajcsi A, Pléh C. Verbal working memory and morphological complexity. Hung J Psychol. 2006;61(2):265–98.

[34] Daneman M, Blennerhassett A. How to assess the listening comprehension skills of prereaders. J Edu Psychol. 1984;76:1372–81.

[35] Janacsek K, Tánczos T, Mészáros T, Németh D. The Hungarian version of listening Span task. Hung J Psychol. 2009;64(2):385–406.

[36] Racsmány M, Lukács Á, Németh D, Pléh C. Hungarian diagnostic tools of verbal working memory functions. Hung J Psychol. 2005;60(4):479–506.

[37] Wechsler D. Wechsler Memory Scale WAIS. 3rd ed. San Antonio, TX: Psychological Corporation; 1997.

[38] Berg EA. A simple objective technique for measuring flexibility in thinking. J Gen Psychol. 1948;39:15–22. 18889466 10.1080/00221309.1948.9918159

[39] Kopp B, Lange F, Steinke A. The reliability of the Wisconsin Card sorting test in clinical practice. Assessment. 2021;28(1):248–63. 31375035 10.1177/1073191119866257 PMC7780274

[40] Tánczos T, Janacsek K, Németh D. Verbal fluency tasks I. Investigation of the Hungarian version of the letter fluency task between 5 and 89 years of age. I. Psych Hung. 2014/a; 29(2): 158–180. 25041746

[41] Tánczos T, Janacsek K, Németh D. Verbal fluency tasks II. Investigation of the Hungarian version of the semantic fluency task between 5 and 89 years of age. Psych Hung. 2014/b; 29(2): 180–207. 25041746

[42] Kroenke K, Spitzer RL, Williams JBW. The PHQ-9: validity of a brief depression severity measure. J Gen Intern Med. 2001;16(9):606–13. 11556941 10.1046/j.1525-1497.2001.016009606.x PMC1495268

[43] Beck AT, Brown G, Epstein N, Steer RA. An inventory for measuring clinical anxiety: psychometric properties. J Consult Clin Psychol. 1988;56(6):893–7. 3204199 10.1037//0022-006x.56.6.893

[44] Perczel-Forintos D, Ajtay Gy B, Cs. Kiss zs, Komlósi S. Kérdőívek, Becslőskálák a Klinikai Pszichológiában. Semmelweis Kiadó; 2018.

[45] Susánszky É, Konkoly-Thege B, Stauder A, Kopp M. Validation of the short (5-item) version of the WHO well-being scale based on a Hungarian representative health survey (Hungarostudy 2002). J Ment Health Psychosom. 2006;7(3):247–55.

[46] WHO. Wellbeing Measures in Primary Health Care/The Depcare Project. WHO Regional Office for Europe; 1998.

[47] Buysse DJ, Reynolds CF 3rd, Monk TH, Berman SR, Kupfer DJ. The Pittsburgh Sleep Quality Index: a new instrument for psychiatric practice and research. Psych Res. 1989;28:193–213. 10.1016/0165-1781(89)90047-4 2748771

[48] Takács J, Bódizs R, Ujma PP, Horváth K, Rajna P, Harmat L. Reliability and validity of the Hungarian version of the Pittsburgh Sleep Quality Index (PSQI-HUN): comparing psychiatric patients with control subjects. Sleep. 2016;20(3):1045–51. 10.1007/s11325-016-1347-7 27115528

[49] Ağargün MY, Kara H, Bilici M, Çilli AS, Telci M, Semiz ÜB, Başoğlu C. The Van dream anxiety scale: a subjective measure of dream anxiety in nightmare sufferers. Sleep Hypn. 1999;1(4):204–11.

[50] Simor P, Kovács I, Vargha A, Csóka Sz, Mengel B, Bódizs R. Nightmares, dream anxiety and psychopathology: the validation of the Hungarian version of the Van anxiety scale. Psych Hung. 2009;24(6):428–38. 20190359

[51] Meijman TF, Vries-Griever AD, Vries GD, Kampman R. The evaluation of the Groningen sleep quality scale (HB 88-13-EX). Groningen: RUG; 1988.

[52] Simor P, Köteles F, Bódizs R, Bárdos G. A questionnaire based study of subjective sleep quality: the psychometric evaluation of the Hungarian version of the Groningen Sleep Quality Scale. J Ment Health Psychosom. 2009;10:249–61.

[53] Johns MWA. New Method for Measuring Daytime Sleepiness: the Epworth Sleepiness Scale. Sleep. 1991;14(6):540–5. 1798888 10.1093/sleep/14.6.540

[54] Soldatos CR, Dikeos DG, Paparrigopoulos TJ. The diagnostic validity of the Athens Insomnia Scale. J Psychosom Res. 2003;55(3):263–7. 12932801 10.1016/s0022-3999(02)00604-9

[55] McDonald JH. Handbook of Biological statistics. 3rd ed. Baltimore, Maryland: Sparky House Publishing; 2014.

[56] Freund RJ, Wilson WJ, Mohr DL. Statistical methods. 3rd ed. Elsevier Science & Technology; 2010.

[5] Chin J, Oh KJ, Seo SW, Na DL. Are depressive symptomatology and self-focused attention associated with subjective memory impairment in older adults? Int Psychogeriat. 2014;26(4):573–80. 10.1017/S104161021300241X 24411288

[58] Corti EJ, Gasson N, Grant H, Wisniewski B, Loftus AM. The multifactorial memory questionnaire and quality of life: a longitudinal study in Parkinson’s Disease. Brain Sci. 2025;15(1):66. 39851433 10.3390/brainsci15010066 PMC11764281

[59] Zuur AF, Ieno EN, Elphick CS. A protocol for data exploration to avoid common statistical problems: data exploration. Methods Ecol Evol. 2010;1(1):3–14.

[60] The jamovi project. Version 2.2.5 [Computer Software]. Retrieved from https://www.jamovi.org. 2021

[61] Jopp D, Hertzog C. Activities, self-referent memory beliefs, and cognitive performance: evidence for direct and mediated relations. Psychol Aging. 2007;22:811–25. 18179299 10.1037/0882-7974.22.4.811

[62] Balash Y, Mordechovich M, Shabtai H, Giladi N, Gurevich T, Korczyn AD. Subjective memory complaints in elders: depression, anxiety, or cognitive decline? Acta Neurol Scand. 2013;127(5):344–50. 23215819 10.1111/ane.12038

[63] Zlatar ZZ, Moore RC, Palmer BW, Thompson WK, Jeste DV. (2014). Cognitive complaints correlate with depression ratherthan concurrent objective cognitive impairment in the successful aging evaluation baseline sample. Journal of Geriatric Psych and Neurol. 2014; 27(3): 181–187. 10.1177/0891988714524628 PMC4255945 24614203

[64] Bhang I, Mogle JA, Hill N, Whitaker EB, Bhargava S. Examining the temporal associations between self-reported memory problems and depressive symptoms in older adults. Aging Ment Health. 2020;24(11):1864–71. 31379193 10.1080/13607863.2019.1647135 PMC7000302

[65] Gopi Y, Madan CR. (2024). Subjective memory measures: Metamemory questionnaires currently in use. Quart J Exp Psychol. 2024; 77(5): 924–942. 10.1177/17470218231183855 PMC11032637 37300278

[66] Mogle JA, Muñoz E, Hill NL, Smyth JM, Sliwinski MJ. Daily memory lapses in adults: characterization and influence on affect. J Gerontol: Ser B. 2019;74(1):59–68. 10.1093/geronb/gbx012 PMC6941205 28329832

[67] Jonker C, Geerlings MI, Schmand B. Are memory complaints predictive for dementia? A review of clinical and population-based studies. Int J Geriat Psych. 2000;15(11):983–91. 10.1002/1099-1166(200011)15:11<983::aid-gps238>3.0.co;2-5 11113976

[68] Carrigan N, Barkus E. A systematic review of cognitive failures in daily life: healthy populations. Neurosci Biobehavioral Rev. 2016;63:29–42. 10.1016/j.neubiorev.2016.01.010 26835660

[69] Pearman A. The interpersonal context of memory complaints. J Appl Geront. 2021;40(11):1601–10. 10.1177/0733464820970065 PMC8099923 33155501

[70] Mecacci L, Righi S, Rocchetti G. Cognitive failures and circadian typology. Personal Individual Diff. 2004;37(1):107–13.

[71] Norman AL, Woodard JL, Calamari JE, Gross EZ, Pontarelli N, Socha J, DeJong B, Armstrong K. The fear of Alzheimer’s disease: mediating effects of anxiety on subjective memory complaints. Aging Ment Health. 2020;24(2):308–14. 30411628 10.1080/13607863.2018.1534081

[72] Lim J, Dinges DF. A meta-analysis of the impact of short-term sleep deprivation on cognitive variables. Psychol Bull. 2010;136(3):375–89. 20438143 10.1037/a0018883 PMC3290659

[73] Fortier-Brochu É, Beaulieu-Bonneau S, Ivers H, Morin CM. Insomnia and daytime cognitive performance: a meta-analysis. Sleep Med Rev. 2012;16(1):84–94. 10.1016/j.smrv.2011.03.008 21636297

[74] Drummond SPA, Walker M, Almklov E, Campos M, Anderson DE, Straus LD. Neural correlates of working memory performance in primary insomnia. Sleep. 2013;36(9):1307–16. 23997363 10.5665/sleep.2952 PMC3738039

[75] Benito-León J, Mitchell AJ, Vega S, Bermejo-Pareja F. A population-based study of cognitive function in older people with subjective memory complaints. J Alzheimer’s Dis. 2010;22:159–70. 20847410 10.3233/JAD-2010-100972

[76] Chen Q, Wu S, Li X, Sun Y, Chen W, Lu J, Zhang W, Liu J, Qing Z, Nedelska Z, Hort J, Zhang X, Zhang B. Basal forebrain atrophy is associated with allocentric navigation deficits in subjective cognitive decline. Front Aging Neurosci. 2021;13:596025. 33658916 10.3389/fnagi.2021.596025 PMC7917187

[77] Rouch I, Anterion CT, Dauphinot V, Kerleroux J, Roche F, Barthelemy JC, Laurent B. Cognitive complaints, neuropsychological performance and affective disorders in elderly community residents. Disabil Rehab. 2008;30(23):1794–802. 10.1080/09638280701667825 18608410

[78] Wang X, Wang Z, Hu H, Qu Y, Wang M, Shen X, Xu W, Dong Q, Tan L, Jt Y. Association of subjective cognitive decline with risk of cognitive impairment and dementia: a systematic review and meta-analysis of prospective longitudinal studies. J Prevent Alzheimer’s Dis. 2021;8(3):277–85.10.14283/jpad.2021.2734101784

[79] Burmester B, Leathem J, Merrick P. Subjective cognitive complaints and objective cognitive function in aging: a systematic review and Meta-analysis of recent cross-sectional findings. Neuropsychol Rev. 2016;26:376–93. 27714573 10.1007/s11065-016-9332-2

[80] Crumley JJ, Stetler CA, Horhota M. Examining the relationship between subjective and objective memory performance in older adults: a meta-analysis. Psychol Aging. 2014;29(2):250–63. 24955993 10.1037/a0035908

[81] Zhou C, Fares BJ, Thériault K, Trinh B, Joseph M, Jauhal T, Sheppard C, Labelle PR, Krishnan A, Rabin L. Vanessa, V. subjective cognitive decline and objective cognitive performance in older adults: a systematic review of longitudinal and cross-sectional studies. J Neuropychol. 2024;00:1–17. 10.1111/jnp.12384 PMC11891377 39075723

[82] Hall KE, Isaac CL, Harris P. Memory complaints in epilepsy: an accurate reflection of memory impairment or an indicator of poor adjustment? A review of the literature. Clin Psychol Rev. 2009;29(4):354–67. 19346044 10.1016/j.cpr.2009.03.001

[83] Ossher L, Flegal KE, Lustig C. Everyday memory errors in older adults. Aging Neuropsychol Cogn. 2013;20(2):220–42. 10.1080/13825585.2012.690365 PMC3443516 22694275

[84] Sander AM, Clark AN, van Veldhoven LM, Hanks R, Hart T, Leon Novelo L, Ngan E, Arciniegas DB. Factor analysis of the everyday memory questionnaire in persons with traumatic brain injury. Clin Neuropsychol. 2018;32(3):495–509. 28849703 10.1080/13854046.2017.1368714

[85] Schmidt IW, Berg IJ, Deelman BG. Relations between subjective evaluations of memory and objective memory performance. Percep Motor Skills. 2001;93(3):761–76. 10.2466/pms.2001.93.3.761 11806600

[86] van der Werf SP, Geurts S, de Werd MME. Subjective memory ability and long-term forgetting in patients referred for Neuropsychological Assessment. Front Neuropsy. 2016;7:605–703. 10.3389/fpsyg.2016.00605 PMC4852420 27199838

[87] Radnan MJ, Nicholson R, Brookman R, Harris CB. Memory compensation strategies in everyday life: similarities and differences between younger and older adults. Sci Rep. 2023;13:8404. 37225766 10.1038/s41598-023-34815-3 PMC10209053

[88] Pearman A, Hertzog C, Gerstorf D. Little evidence for links between memory complaints and memory performance in very old age: longitudinal analyses from the Berlin Aging Study. Psychol Aging. 2014;29(4):828–42. 25089853 10.1037/a0037141

[89] Bouazzaoui B, Fay S, Guerrero-Sastoque L, Semaine M, Isingrini M, Taconnat L. Memory age-based stereotype threat: role of locus of control and anxiety. Exp Aging Res. 2020;46(1):39–51. 31752597 10.1080/0361073X.2019.1693009

[90] Illman NA, Moulin CJA, Kemp S. Assessment of everyday memory functioning in temporal lobe epilepsy and healthy adults using the multifactorial memory questionnaire (MMQ). Epilepsy Res. 2015;113:86–9. 25986194 10.1016/j.eplepsyres.2015.03.011

[91] Cavallini E, Bottiroli S, Fastame MC, Hertzog C. Age and subcultural differences on personal and general beliefs about memory. J Aging Stud. 2013;27(1):71–81. 23273559 10.1016/j.jaging.2012.11.002

[92] Fritsch T, McClendon MJ, Wallendal MS, Hyde TF, Larsen JD. Prevalence and cognitive bases of subjective memory complaints in older adults: evidence from a community sample. J Neurodegen Dis. 2014; 176843. 10.1155/2014/176843 PMC4437337 26317004

[93] Van Bergen S, Brands I, Jelicic M, Merckelbach H. Assessing trait memory distrust: psychometric properties of the squire subjective memory questionnaire. Legal Criminolog Psychol. 2010;15(2):373–84.

[94] Van Bergen S, Jelicic M, Merckelbach H. (2009). Are subjective memory problems related to suggestibility, compliance, false memories, and objective memory performance? The Am J Psychol. 2009; 122(2): 249–257. 19507430

[95] Lozoya-Delgado P, Ruiz-Sánchez de León JM, Pedrero-Pérez E. Validation of the cognitive complaints’ questionnaire for young adults: relationship between subjective memory complaints, prefrontal symptomatology and perceived stress. Revista Neurol. 2012;54:137–50. 22278890

[96] Molina-Rodriguez S, Pellicer-Porcar O, Mirete-Fructuoso M. Perceived stress and subjective memory complaints in young adults: the mediating role of the executive functions. Revista De Neurol. 2018;67(3):84–90. 29999172

[97] de Ruiz-Sánchez JM, Llanero-Luque M, Lozoya-Delgado P, Fernández-Blázquez MA, Pedrero-Pérez EJ. Neuropsychological study of young adults with subjective memory complaints: involvement of the executive functions and other as-sociated frontal symptoms. Revista Neurol. 2010;51(11):650–60. 21108227

[98] Baddeley AD. Fractionating the central executive. In: Stuss DT, Knight RT, editors. Principles of frontal lobe function. New York: Oxford University Press; 2002. pp. 246–60.

[99] Hester R, Garavan H. Working memory and executive function: the influence of content and load on the control of attention. Mem Cogn. 2005;33:221–33. 10.3758/bf03195311 16028577

[100] Kane MJ, Bleckley MK, Conway AR, Engle R. W. A controlled-attention view of working-memory capacity. J Exp Psychol: Gen. 2001;130:169–83. 11409097 10.1037//0096-3445.130.2.169

[101] Kane MJ, Engle RW. Working memory capacity and the control of attention: the contribution of goal neglect, response competition, and task set to Stroop interference. J Exp Psychol: Gen. 2003;132:47–70. 12656297 10.1037/0096-3445.132.1.47

[102] Colvin LE, Malgaroli M, Chapman S, MacKay-Brandt A, Cosentino S. Mood and personality characteristics are associated with metamemory knowledge accuracy in a community-based cohort of older adults. J Internat Neuropsychol Soc: JINS. 2018;24(5):498. 10.1017/S1355617717001345 PMC8082693 29400264

[103] Munoz E, Sliwinski MJ, Smyth JM, Almeida DM, King HA. Intrusive thoughts mediate the Association between Neuroticism and cognitive function. Personal Ind Diff. 2013;55(8):898–903. 10.1016/j.paid.2013.07.019 PMC3965708 24683284

[104] Crow A. Associations between Neuroticism and executive function outcomes: response inhibition and sustained attention on a continuous performance test. Percep Motor Skills. 2019;126(4):623–38. 10.1177/0031512519848221 31146642

[105] Kane MJ, Gross GM, Chun CA, Smeekens BA, Meier ME, Silvia PJ, Kwapil TR. For whom the Mind Wanders, and when, Varies Across Laboratory and Daily-Life settings. Psychol Sci. 2017;28(9):1271–89. 28719760 10.1177/0956797617706086 PMC5591044

[106] Robison MK, Gath KI, Unsworth N. The neurotic wandering mind: an individual differences investigation of neuroticism, mind-wandering, and executive control. Q J Exp Psychol. 2017;70(4):649–63. 10.1080/17470218.2016.1145706 26821933

[107] van der Werf SP, Vos SH. 2011. Memory Worries and Self-Reported Daily Forgetfulness: A Psychometric Evaluation of the Dutch Translation of the Multifactorial Memory Questionnaire. The Clin Neuropsychol. 2011; 25(2): 244–268. 10.1080/13854046.2010.543290 21253959

